# Supplementary material for: The Effects of Downhill Running and Maturation on Histological and Morphological Properties of Tendon and Enthesis in Mice
Source: Biology (Basel). 2023 Mar 16;12(3):456. doi: 10.3390/biology12030456 (PMC10045940; doi:10.3390/biology12030456)
Supplement: Supplementary file 1 [file biology-12-00456-s001.zip › biology-2063362-supplementary.pdf]

Title: The effects of downhill running and maturation on histological and morphological properties of tendon and enthesis in mice

Supplementary methods

Molecular biology methods

Real-time PCR was used to quantify the target genes expressed in tendons and enthesis FC at the mRNA level. After micro-CT imaging, they were infiltrated into the RNAlater^{TM} Stabilization Solution (Invitrogen) again as soon as possible. The tendon and FC areas were separated using a microsurgical knife under a stereomicroscope on the same day that the micro-CT imaging was performed. The samples were carefully collected with observation under a stereomicroscope to ensure that no bone marrow was included. During analysis, each sample was homogenized in ISOGEN (NIPPON GENE, Tokyo, Japan). Chloroform was added dropwise; after centrifugation (12,000 ×*g*, 15 min, 4 °C), only the aqueous layer was extracted. Isopropanol was added dropwise, and centrifugation was performed again (12,000 ×*g*, 10 min, 4 °C). After washing with 70% ethanol, centrifugation was performed (7,500 ×*g*, 5 min, 4 °C), and the solution was dissolved in UltraPure™ DEPC-Treated Water (Thermo Fisher Scientific) to prepare the total RNA solution. To remove genomic DNA, recombinant DNAse I (TaKaRa Biotechnology, Shiga,

Japan) and recombinant RNase inhibitor (TaKaRa Biotechnology) were added to the total RNA solution and incubated at 37 °C for 30 min. Agencourt RNA CleanXP (Beckman Coulter, Brea, CA, USA) was added, and the total RNA solution was purified on a magnetic plate. A NanoDrop Lite UV-Vis Spectrophotometer (Thermo Fisher Scientific) was used to measure the purity and concentration of RNA in the purified samples. The OD ratio (A260/A280) of all RNA samples was confirmed to be within 1.8–2.0. After concentration measurement, cDNA was synthesized using a High-Capacity cDNA Reverse Transcription kit (Applied Biosystems). cDNA was synthesized under the following conditions: 10, 120, and 10 min at 25, 37, and 85 °C, respectively. Real-time PCR was performed using the StepOnePlus system (Applied Biosystems) and TaqMan Gene Expression Assay probe (Applied Biosystems). PCR was performed under the following conditions: 20 s at 95 °C and 20 s at 60 °C, with the cycle repeated 40 times. The target primer was as follows: *Tnf-a* (Mm00443258-m1), and *Il-6* (Mm00446190-m1). *Hypoxanthine phosphoribosyltransferase 1* (*Hprt1*; Mm00446968_m1) was used as a reference gene. The target gene transcript levels normalized to *Hprt1* were calculated using the $2^{-\Delta\Delta Ct}$ equation.
